# Comparative Study of the Genetic and Biochemical Variability of *Polyscias filicifolia* (Araliaceae) Regenerants Obtained by Indirect and Direct Somatic Embryogenesis as a Source of Triterpenes

**DOI:** 10.3390/ijms22115752

**Published:** 2021-05-27

**Authors:** Anita A. Śliwińska, Agnieszka Białek, Renata Orłowska, Dariusz Mańkowski, Katarzyna Sykłowska-Baranek, Agnieszka Pietrosiuk

**Affiliations:** 1 Department of Pharmaceutical Biology and Medicinal Plant Biotechnology, Faculty of Pharmacy, Medical University of Warsaw, Banacha 1, 02-097 Warsaw, Poland; asliwinska@wum.edu.pl (A.A.Ś.); agnieszka.pietrosiuk@wum.edu.pl (A.P.); 2 Department of Biotechnology and Nutrigenomics, Institute of Genetics and Animal Biotechnology, Polish Academy of Sciences, Postępu 36 A, Jastrzębiec, 05-552 Magdalenka, Poland; a.bialek@ighz.pl; 3 Plant Breeding and Acclimatization Institute-National Research Institute, Radzików, 05-870 Błonie, Poland; r.orlowska@ihar.edu.pl (R.O.); d.mankowski@ihar.edu.pl (D.M.)

**Keywords:** somatic embryogenesis, somaclonal variation, AFLP, metAFLP, triterpenoids, Araliaceae

## Abstract

*Polyscias filicifolia* (Araliaceae) is broadly used in traditional medicine in Southeast Asia due to its antimicrobial, immunomodulating and cytotoxic activities. The main groups of compounds responsible for pharmacological effects are believed to be oleanolic triterpene saponins. However, *Polyscias* plants demonstrate relatively slow growth in natural conditions, which led to applying a developing sustainable source of plant material via primary (PSE), secondary (DSE) and direct somatic embryogenesis from DSE (TSE). The AFLP and metAFLP genotyping resulted in 1277 markers, amplified by a total of 24 pairs of selective primers. Only 3.13% of the markers were polymorphic. The analysis of variance showed that the PSE and TSE regenerants differed only in terms of root number, while the DSE plantlets differed for all studied morphological characteristics. Further, the chemical analysis revealed that oleanolic acid (439.72 µg/g DW), ursolic acid (111.85 µg/g DW) and hederagenin (19.07 µg/g DW) were determined in TSE regenerants. Our results indicate that direct somatic embryogenesis ensures the production of homogeneous plant material, which can serve as a potential source of triterpene compounds. Plants obtained via somatic embryogenesis could also be reintroduced into the natural environment to protect and preserve its biodiversity.

## 1. Introduction

*Polyscias filicifolia* (C. Moore ex E. Fourn) L.H. Bailey is a representative of the Araliaceae family. *P. filicifolia* is a species that shares the genus *Polyscias* with between 116 [1] and 150 others [2]. It is an evergreen, six-meter-tall shrub that naturally occurs in Southeast Asia, but is also available as a decorative potted plant [3]. *Polyscias* spp. extracts are known to possess a number of pharmacological properties, including antimicrobial, immunostimulant and cytotoxic activities [reviewed in 1]. Approximately 97 compounds of different chemical classes have been identified in selected *Polyscias* species; however, the main groups of compounds responsible for pharmacological effects are believed to be oleanolic triterpene saponins, present in the leaves and roots, and polyacetylenes, which only occur in the roots [4,5]. *Polyscias* spp. extracts may also contain polyphenols, flavonoids, glycosides, saponins, cyanogenic glycosides, sterols and alkaloids [1,4,6,7]. In Southeast Asia, plants of *Polyscias* genus are broadly used in traditional medicine; for example, it is recorded in the official Vietnamese Pharmacopoeia as an anti-fatigue and cardiac drug [8].

As *Polyscias* plants demonstrate relatively slow growth in natural conditions, and their natural range is limited to tropical and subtropical areas, there is a need for biotechnological approaches to provide a more sustainable source of plant material. Śliwińska et al. [3] developed an efficient method of micropropagation of *P. filicifolia* via somatic embryogenesis from the apical meristems of the shoots. In addition, polyphenolic compound production was found to be enhanced in the shoot cultures treated with elicitors such as salicylic acid (SA) and/or methyl jasmonate (MeJA) [9]. In addition, shoot extracts derived from in vitro cultures were found to reduce cancer cell viability and increase their mortality, and to exert a protective effect on healthy cells. In subsequent investigations, shoot extracts of *P. filicifolia* derived from in vitro cultures also exhibited anti-genotoxic and anti-photogenotoxic activities [10].

In vitro culture could serve as a reliable source of plant material for diverse applications in science and industry, thus protecting natural resources and ensuring reproducibility of desired specific genetic and physiological features. For many medicinal and endangered plants, various methods of regeneration have been described [11,12,13]. The most frequently used methods of plant regeneration are somatic embryogenesis and organogenesis. The former allows a vast number of regenerants to be obtained; however, it may result in genetic changes such as chromosome breakage and rearrangement, increased ploidy or the exchange of bases in the DNA sequence [11,13,14,15,16]. It is also characterized by a greater chance of epigenetic modifications, e.g., at the DNA methylation level [17,18]. Moreover, epigenetic reprogramming of embryogenic cells may result in hereditable, but potentially reversible changes, leading to chemical modification of DNA and histone proteins, including chromatin remodeling. It is believed that although epigenetic changes are not encoded by DNA, they can still be passed on to the next generation [13,19].

Various markers such as Simple Sequence Repeat (SSR), Randomly Amplified Polymorphic DNA (RAPD), Restriction Fragment Length Polymorphism (RFLP), Inter Simple Sequence Repeats (ISSR) and Amplified Fragment Length Polymorphism (AFLP) are used to assess the homogeneity of plant material obtained by somatic embryogenesis and/or organogenesis [20]. Of these markers, AFLP is recommended because it allows a large number of polymorphisms distributed throughout the genome to be tested; it is also a relatively easy method, and requires only small amounts of DNA. This method has been used for the detection of somaclonal variants, among others [21,22,23,24,25].

Other methods for detecting changes at the genetic and epigenetic level are Methylation-Sensitive Amplification Polymorphism (MSAP) [20] and Methylation-Sensitive Amplified Fragment Length Polymorphism (metAFLP) [26]; the latter allows simultaneous analysis of changes in genetic, i.e., in DNA base sequence, and epigenetic, i.e., base methylation, properties. This method is based on the action of two restriction enzymes which differ in their sensitivity to DNA methylation at the recognized site: *Acc*65I, which is sensitive to methylation, and *Kpn*I, which is not. Based on the resulting metAFLP profiles produced by the two enzymes (*Kpn*I/*Mse*I and *Acc*65I/*Mse*I), it is possible to identify methylation; in addition, the *Kpn*I/*Mse*I enzymes can determine variation at the sequential level.

The AFLP profiles produced by metAFLP can be used to identify somaclonal changes induced by in vitro cultures and calculate quantitative characteristics. This technique has been used to identify somaclonal variation of *Hordeum vulgare* [18,26], *Gentiana pannonica* [27], *Gentiana cruciata* [28], Triticale (x *Triticosecale* spp.) regenerants [29,30] and *Arabidopsis thaliana* regenerants [31].

This is the first study to assess the morphological, genetic and epigenetic variation of *Polyscias filicifolia* plants derived by three regeneration methods: indirect somatic embryogenesis (PSE), direct somatic embryogenesis from primary somatic embryos (DSE) and direct somatic embryogenesis from secondary somatic embryogenesis (TSE). Genetic and epigenetic variations were investigated using AFLP and metAFLP. Further, biochemical variation of resulting regenerants cultivated in vitro was studied by the analysis of the triterpenoids oleanolic acid, ursolic acid and hederagenin and was compared to nonembryogenic and embryogenic callus tissues, TSE regenerants cultivated ex vitro as well as donor plant using gas chromatography.

## 2. Results

### 2.1. Comparison of Regenerant Morphology

**Figure 1:**
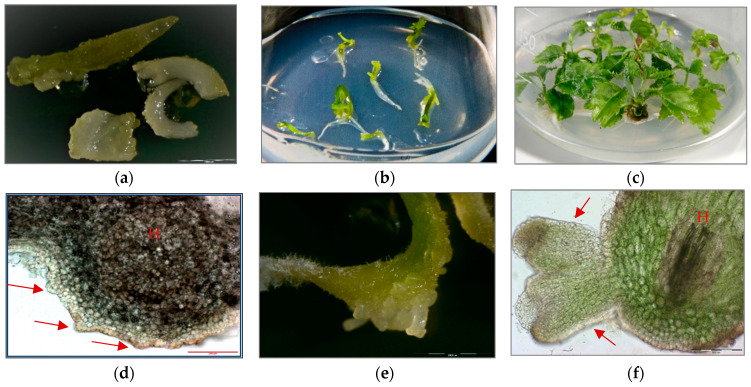

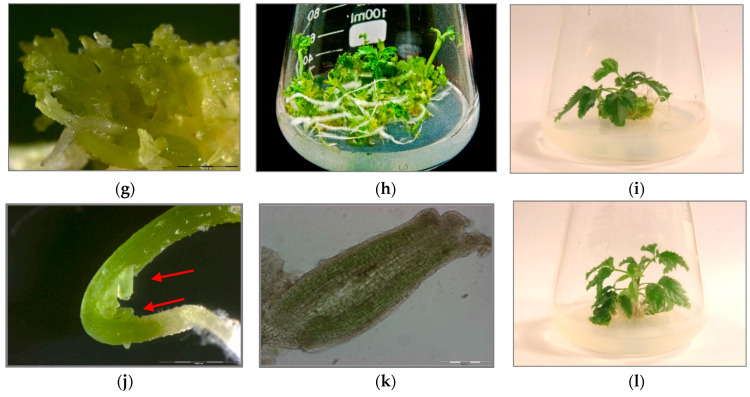
(**a**) Primary somatic embryos (PSE) of *Polyscias filicifolia* at different stages obtained after transferring embryogenic callus into hormone-free ½ Murashige and Skoog (MS) liquid medium within six-week passage (bar = 2000 µm). **(b**) Conversion of PSE into plantlets after transfer on Nitsch and Nitsch medium (NN) solid medium supplemented with 0.5 mg/L kinetin (KIN), 0.1 mg/L indole-3-butyric acid (IBA) and 10 mg/L adenine sulfate (Ads). (**c**) Plantlets developed from germinating PSE after four-week culture. (**d**) Secondary somatic embryos (DSE) formed directly on the hypocotyl (H) of the PSE seven days after transferring onto hormone-free ½ MS solid medium and 15 g/L sucrose (cross-section) (bar = 200 µm). (**e**) DSE at different developmental stages: globular, heart-shaped, torpedo-shaped formed after three weeks of cultivation on hormone-free ½ MS solid medium and 15 g/L sucrose (bar = 2000 µm). (**f**) Cross-section of DSE in the torpedo stage formed on the hypocotyl part (H) of PSE after three weeks of culture on hormone-free ½ MS solid medium and 15 g/L sucrose (bar = 200 µm). (**g**) DSE germinating into plantlets after two weeks of culture on hormone-free ½ MS solid medium and 15 g/L sucrose (bar = 2000 µm). (**h**) Development of plantlets obtained from DSE after four weeks of culture on hormone-free ½ MS solid medium and 15 g/L sucrose. (**i**) Plantlet developed from DSE after four weeks of culture on NN solid medium supplemented with 0.5 mg/L KIN, 0.1 mg/L IBA and 10 mg/L Ads. (**j**) Development of TSE embryos directly on the hypocotyls of DSE two weeks after transfer to hormone-free ½ MS solid medium and 15 g/L sucrose (bar = 2000 µm). (**k**) Cross-section of a TSE embryo in the early cotyledonous stage obtained on hypocotyl of DSE after three weeks of culture on hormone-free ½ MS solid medium and 15 g/L sucrose (bar = 200 µm). (**l**) Plantlet regenerated from TSE after four weeks of culture on NN solid medium supplemented with 0.5 mg/L KIN, 0.1 mg/L IBA and 10 mg/L Ads.

Three sets of *P. filicifolia* regenerants were investigated: obtained through indirect somatic embryogenesis (PSE) from callus tissue; secondary somatic embryos (DSE) induced on the hypocotyls of primary somatic embryos (PSE); and tertiary somatic embryos obtained through direct somatic embryogenesis from DSE hypocotyl region (TSE) (Figure 1).

Among the three sets of regenerants, the TSE group varied significantly (*p* < 0.05) in respect of investigated morphological traits. TSE regenerants had the shortest shoots and the smallest leaves, and formed the smallest number of leaves and roots (Table 1).

**Figure 2:**
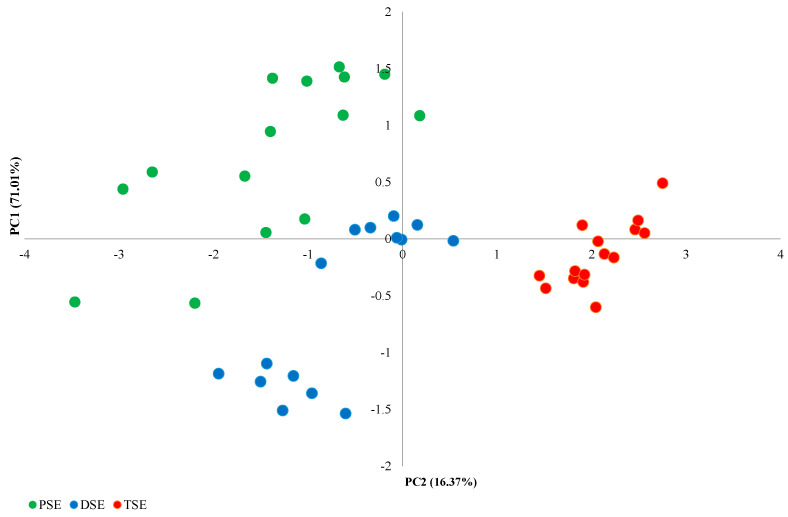
PCA analysis of variability between PSE (obtained through indirect somatic embryogenesis), DSE (secondary somatic embryos induced on the hypocotyls of PSE) and TSE (obtained through direct somatic embryogenesis from DSE) sets of regenerates based on morphological traits.

The PCA analysis of the morphological features identified four main components (Figure 1). The first principal component (PC1), comprising the number of leaves and the mean length of the largest leaf, explained 71.01% of the observed variability; this component was the only one to fulfil the eigenvalue criterion (≥1). The second main component (PC2), formed by the height of the plants and the number of roots, explained 16.37% of the observed variability (Figure 2). The PSE, DSE and TSE regenerants formed separate clusters; in addition, PSE and DSE were more diverse than TSE.

### 2.2. AFLP and metAFLP Analysis

**Figure 3:**
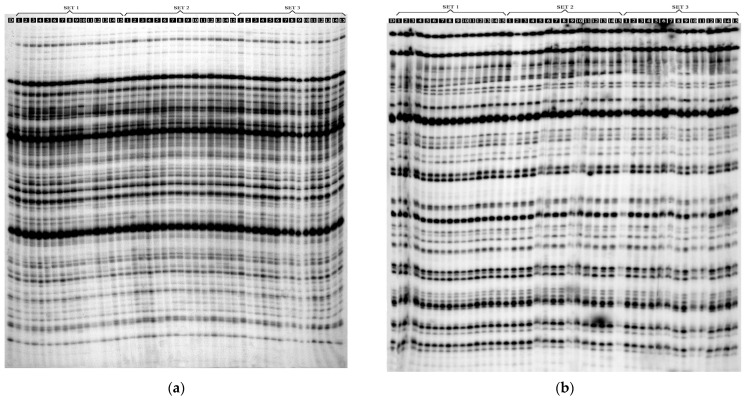
Electrophoretic separation of the: (**a**) amplified AFLP products using *E*-ACT/*M*-CGA, selective primers: (D) donor plant; SET 1: (PSE1-PSE15)—PSE regenerants; SET 2: DSE1-DSE15—DSE regenerants; SET 3: TSE1–TSE15—TSE regenerants; (**b**) amplified metAFLP products using CpXpG-AAG/M-CGC selective primers; genomic DNA was digested with *Kpn*I/*Mse*I: (D) donor plant; SET 1: (PSE1-PSE15)—PSE regenerants; SET 2: DSE1-DSE15—DSE regenerants; SET 3: TSE1–TSE15—TSE regenerants.

The AFLP method using EcoRI and MseI allowed the identification of 428 DNA fragments, amplified using eight pairs of selective primers. In total, 1.17% of the polymorphic bands were evaluated. Individual pairs of selective primers replicated between 34 (E-AAG/M-CAC) and 68 (E-ATC/M-CGA) DNA fragments (Table 2). The mean number of DNA fragments amplified by a single pair of selective AFLP primers was 53.5. The number of polymorphic bands ranged from one to three, depending on the selective primer system. Polymorphic bands were found in the following selective primer pairs: E-ACT/M-CGA; E-AGG/M-CTT; E-ATC/M-CGA (Table 2 and Figure 3).

The metAFLP method enabled the identification of 422 DNA fragments using KpnI/MseI restriction endonucleases and 427 using Acc65I/MseI pairs. A single pair of primers replicated an average of 52.7 (KpnI/MseI) and 53.3 DNA fragments (Acc65I/MseI). The largest numbers of DNA fragments, i.e., 66 and 65, were obtained for the CpXpG-AAG/M-CGC pair of selective primers. For the KpnI/MseI restriction endonuclease pair, a total of 20 polymorphic DNA fragments (4.74%) were found that were observed in all primers except CpXpG-AGG/M-CAC. For the Acc65I/MseI endonuclease pair, a total of 15 polymorphic DNA fragments were found, and they were present in all primer pairs except CpG-TGA/M-CGC, CpGpG-GAG/M-CAC and CpXpG-AGG/M-CAC. For the Acc65I/MseI endonuclease pair, a total of 15 polymorphic DNA fragments (3.51%) were found, and they were present in all primer pairs except CpG-GAG/M-CAC and CpXpG-AAG/M-CAC (Table 2).

For all endonuclease pairs analyzed (EcoRI/MseI, KpnI/MseI, Acc65I/MseI), 1277 DNA monomorphic and polymorphic fragments were identified; of these, only 40 were polymorphic. Thus, the polymorphism among the studied regenerants (PSE, DSE, TSE) and the donor plant (R) was 3.13%.

The analysis of electrophoretic profiles obtained after digestion with three different pairs of restriction enzymes: *Eco*RI/*Mse*I, *Kpn*I/*Mse*I, *Acc*65I/*Mse*I for three sets of plantlets (PSE, DSE, TSE) revealed no specific bands for the *Eco*RI/*Mse*I restriction endonuclease system. In contrast, for the *Kpn*I/*Mse*I platform, two individual bands were found for the PSE regenerants (Table 3). For the *Acc*65I/*Mse*I restriction endonuclease enzyme, one specific band was found in the DSE and TSE sets (Table 3). However, no bands specific to the donor plant were found with any of the three endonuclease systems (Table 4).

**Figure 4:**
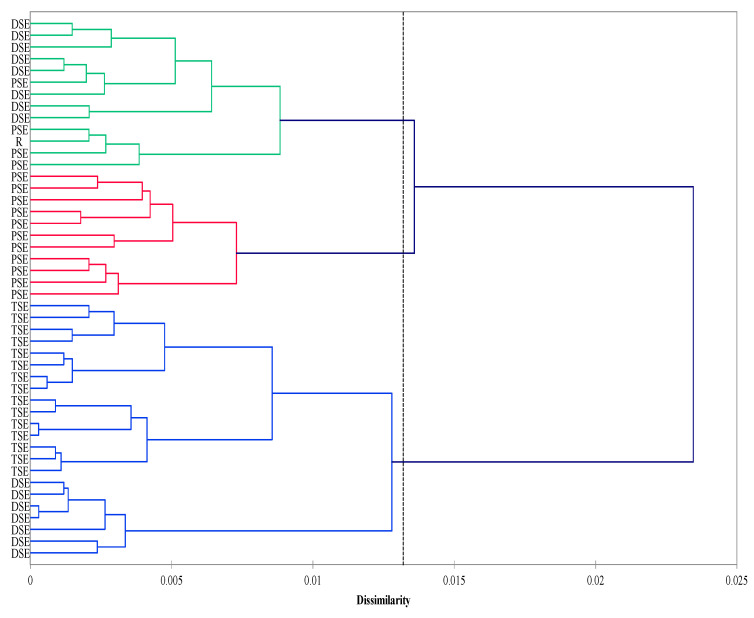
Agglomeration (Ward method; Dice coefficient) analysis of the R, PSE, DSE and TSE sets of regenerates based on AFLP and metAFLP markers. Cluster significance (dotted line) determined on the basis of pseudo-F statistics with significance level *p* < 0.05 (R—donor plant; PSE—somatic embryos obtained through indirect somatic embryogenesis; DSE—secondary somatic embryos induced on the hypocotyls of PSE; and TSE—somatic embryos obtained through direct somatic embryogenesis from DSE).

The agglomeration analysis identified three clusters. The first consisted mainly of the PSE regenerants and donor plants (R). The second cluster was composed of the DSE plantlets, whereas the third included the TSE regenerants and some from the DSE group. The PSE group was less homogeneous than the DSE and TSE groups (Figure 4).

**Figure 5:**
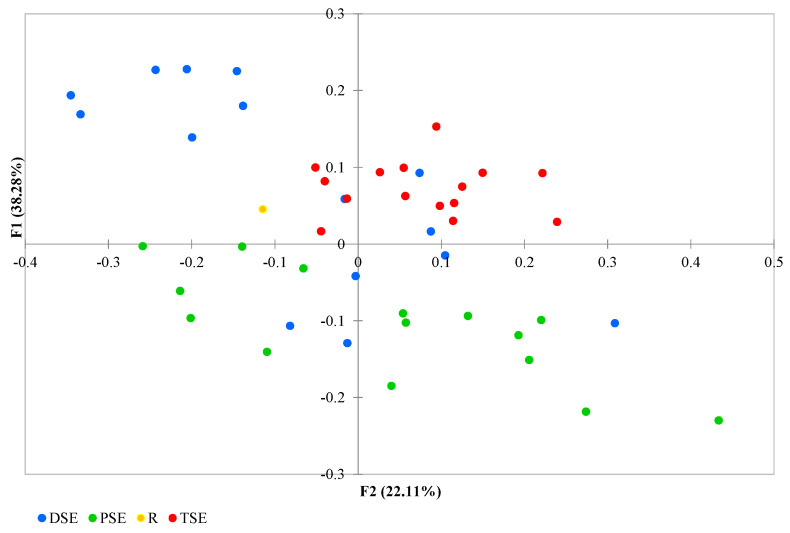
Principal Coordinate Analysis (PCoA) of the R, PSE, DSE and TSE sets of regenerants based on AFLP and metAFLP markers (R—donor plant; PSE—somatic embryos obtained through indirect somatic embryogenesis; DSE—secondary somatic embryos induced on the hypocotyls of PSE; and TSE—somatic embryos obtained through direct somatic embryogenesis from DSE).

The PCoA analysis found coordinates F1 and F2 together to explain 66.29% of the variation: with F1 explaining 38.28% and F2 explaining 22.11% (Figure 5). Some DSE individuals overlapped with samples from the other groups. The group formed of PSE plantlets exhibited a wide range of variability, with the regenerants partly overlapped with a few individuals from the DSE. The TSE samples were less variable than those of the PSE, and their data overlapped with those of some DSE regenerants. The donor plant data lay somewhat in between the groups (Figure 5).

Regarding the epigenetic changes induced in the in vitro regenerants identified by metAFLP, the level of sequence variation appeared to decrease from 1.25 to 0.75% in the order PSE–DSE–TSE. Similarly, an analogous direction of changes was revealed for de novo methylation, ranging from 0.41 to 0.18%, and for the degree of demethylation of restriction sites, increasing from 0.54 to 0.84% (Table S3).

### 2.3. Qualitative and Quantitative Assessment of Triterpenoids in Regenerants

In the first stage of the phytochemical analysis, the most efficient solvent and extraction methods were selected based on the detection and quantification of three aglycones: OA, UA and HE. Among all the tested procedures, the highest concentrations of OA, UA and HE were obtained by extraction with methanol in the Soxhlet apparatus; compared to sonification, this approach obtained more than 43% higher yields of OA, over 55% more UA and over 95% more HE (Table 5).

The sample cleaning method based on SPE Chromabond columns allowed up to 98.34% recovery of OA standard solutions and 77.35% of HE (Table S2).

**Figure 6:**
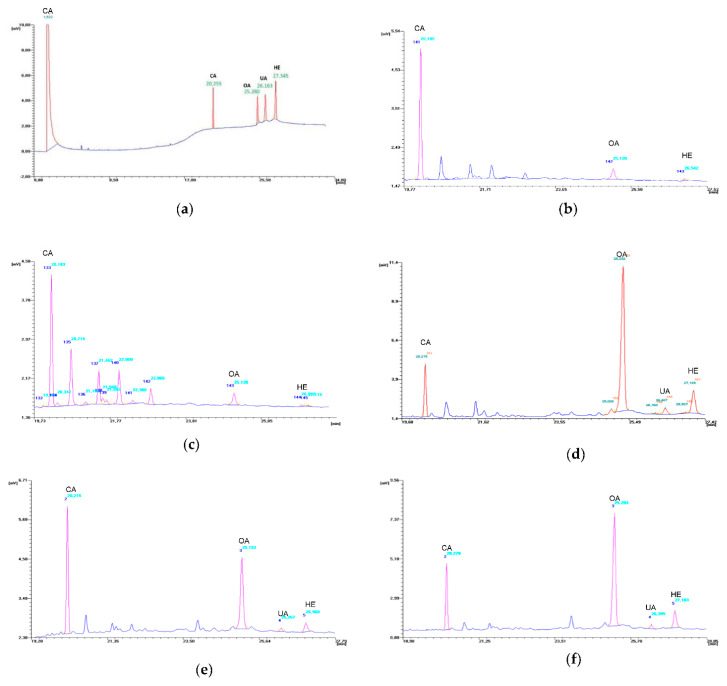
GC chromatograms: (**a**) mixture of the trimethylsilyl derivatives of standard substances: oleanolic acid (OA), ursolic acid (UA), hederagenin (HE) and cholesterol acetate (CA); (**b**)extract of nonembryogenic callus; (**c**) extract of embryogenic callus; (**d**) extract of TSE regenerants; (**e**) extracts of TSE plants ex vitro; (**f**) extract of donor plant.

The conditions for the gas chromatography separation of OA, UA and HE applied in this work appear to be effective (Figure 6), and the obtained standard curves demonstrate high linear correlation coefficients (Table S2).

The phytochemical analysis found the dominant compound in the tested plant material to be OA, with its highest concentration determined in TSE regenerants (439.72 ± 0.64 µg/g DW) (Table 6); this value was also almost eight-fold higher than in donor plants and over nine-fold higher than in TSE regenerants growing ex vitro. The TSE group also demonstrated the highest levels of UA and HE; however, the UA level was almost three-fold higher in donor plants than TSE cultivated in vitro, and UA was not detected in nonembryogenic or embryogenic callus extracts. Similar quantities of HE were determined in the donor plant and ex vitro cultivated regenerants, but these levels were almost seven-fold lower than in TSE regenerants. In addition, the HE content was over 11-fold lower in the nonembryogenic and embryogenic callus tissues than in the TSE group, but only around two-fold lower than in donor plants (Table 6).

## 3. Discussion

Somatic embryogenesis is an alternative, vegetative method of clonal multiplication used in many seed-grown plants that are difficult to propagate, as well as endangered species and those with medicinal properties or are otherwise useful [12,32,33]. As plant regeneration by somatic embryogenesis is an asexual process that only involves mitotic cell division, the expected result is to obtain genetically uniform plants. However, some plants obtained by in vitro culture procedures nevertheless demonstrate some degree of somaclonal variability (SV) due to changes arising in cell culture. Hence, somatic embryogenesis, especially procedures based on indirect somatic embryogenesis, carries the risk of occurrence of phenotypic, genetic and epigenetic changes in the received regenerants.

Therefore, to confirm the efficacy of embryogenesis, it is necessary to conduct a morphological assessment of the regenerants, together with and paying attention to the structural features of the leaves, flowers and fruit. In addition, the plant material should be analyzed at the genetic level to identify changes in DNA sequence. However, as changes in the regenerant genome may also occur following changes in the level of DNA methylation, these should also be confirmed using methylation-sensitive markers [15,21,34,35].

This work describes the first such evaluation of *Polyscias filicifolia*, with the aim of determining the extent to which plant material from a donor plant can change after being introduced into in vitro culture. Our PCA analysis found that the samples from PSE, DSE and TSE groups could be separated into three groups based on methylation levels. The PSE regenerants demonstrated more variation than those from the DSE and TSE groups. This phenomenon could be explained by the fact that PSE regenerants originated from callus tissue which is known to be less genetically stable than organized tissues of somatic embryos, the source of explants for DSE and TSE. This can be attributed to the selection of competent cells involved in embryo formation using a specific genetic program [36,37] and the reduction in variation could be due to the so-called bottleneck effect [14].

In the current study, the molecular data and morphological data are consistent, and suggest that the PSE, DSE and TSE regenerants generally form distinct groups; even so, some degree of variability and intermixing of regenerants was still observed. As molecular markers are not necessarily related to morphological traits or may not reflect genetic (or epigenetic) variation, it is understandable why such markers resulted in less pronounced differentiation between groups. However, the PSE, DSE and TSE were also subjected to molecular assessment using the AFLP method, which has been successfully used in studies of the homogeneity of plants regenerated by somatic embryogenesis and organogenesis in vitro [21,22,23,25,38,39,40].

In the present study, the AFLP analysis was applied using eight pairs of selective primers, which allowed the identification of 428 bands, with polymorphic bands representing only 1.2%. A similarly high level of homogeneity (98.8%) was previously obtained in plant culture obtained by somatic embryogenesis of *Bamboo nutans* [25], where AFLP based on six selective primers led to the identification of 407 bands, with polymorphic bands accounting for 1.17%. AFLP testing has also revealed very high levels of genetic homogeneity in *Fressia hybrida* regenerants [41], as well as in *Coffea arabica* plants for two hybrids, using embryogenic suspension (ESP) and secondary somatic embryogenesis (SCE) as propagation systems [39]. The ESP-derived plants showed no AFLP polymorphism compared to mother plants and ranges of 0.003%.

However, some reports indicate the occurrence of higher levels of genetic variability during the development of somatic embryos. For comparison, an AFLP study of 23 rye regenerants obtained from somatic embryos allowed the identification of polymorphism frequency of 8.8%. High levels of polymorphism were obtained independently in plants from different cell lines, revealing potential mutational hot spots: independent mutational events were found to occur in the same genome regions of these plants [22].

The AFLP method also proved to be a sensitive and reliable molecular marker for detecting possible somaclonal changes in the *Echinacea purpurea* micropropagation system. Research on 40 regenerants obtained from leaf organogenesis and five donor plants, using eight primer pairs, detected 3805 fragments, of which 301 (9.40%) were polymorphic. The mean percentage of polymorphic fragments in the five donor groups ranged from 1.6% to 20.6% [23]. Studies suggest that the variability observed in plants regenerated from somatic embryos may be due to changes in the level of DNA methylation; this process may lead to changes at the epigenetic level, which, unlike genetic changes, do not depend on the DNA sequence and may be responsible for changes in gene function or expression [15,16,18,19]. DNA methylation is one of the basic, hereditary epigenetic features that determine the silencing of specific DNA sequences.

The process of somatic embryogenesis is regulated by many factors, including DNA methylation [16,42,43]. In embryogenic cells, the level of DNA methylation is modified by auxin activity and in vitro conditions; the methylation level in turn affects the expression patterns of the genes involved in SE. In the present study, the *P. filicifolia* regenerants demonstrated a significant range of methylation, which may be associated with culture stress, particularly the addition of auxin, i.e., 2,4-D, and the number of passages, i.e., the duration of culture. A review of the literature shows that 2,4-D, and auxins in general, play an important role in the induction of somatic embryos in many plants, and their use can result in a range of epigenetic and genetic changes in cells, including methylation and mutations in DNA [15,44,45,46]. In addition, the presence of high concentrations of 2,4-D can contribute to numerous disorders in embryo development and disrupt normal genetic and physiological processes in cells [46,47,48]. It has also been shown that 2,4-D and stressful environmental conditions posed by in vitro culture affect the processes of histone modification, chromatin structure rearrangement and DNA methylation, which in turn can contribute to changes in gene expression and thus interfere with the development of somatic embryos [43,45,49]. In the present study, to obtain *P. filicifolia* regenerants by indirect somatic embryogenesis, it was necessary to first induce a nonembryogenic callus using a relatively high concentration of 2,4-D and sucrose (6%).

The present study evaluated the variability of three groups of *P. filicifolia* regenerants, viz. PSE, DSE and TSE, and the donor plant based on the metAFLP method. It allows simultaneous detection of changes in DNA sequence and methylation level based on the properties of *Kpn*I/*Mse*I and *Acc*65I/*Mse*I restriction enzyme pairs, which vary in cytosine methylation sensitivity. In the current study, metAFLP analysis allowed the identification of 849 bands for both pairs of restriction enzymes (*Acc*65I/*Mse*I and *Kpn*I/*Mse*I), with the level of variability found to be 4.74% for the *Kpn*I/*Mse*I enzymes, and 3.51% for the *Acc65*I/*Mse*I enzymes.

metAFLP analysis has also been used to detect epigenetic variability in various regeneration systems of plants grown in vitro, such as in *Hordeum vulgare* [50], as well as *Gentiana cruciata* regenerated from somatic embryos that have previously undergone short-term and long-term cryopreservation [28]. It was also used to examine tissue culture-induced variability of triticale regenerants derived from four different genotypes using androgenesis and somatic embryogenesis [29]. It also identified changes induced by tissue culture conditions, including sequence variations and changes in methylation patterns, in *Gentiana pannonica* plants derived from somatic embryogenesis; in this case, tissue culture-induced variation was found to be 16% [27]. The metAFLP method was also effective at detecting variability at the DNA sequence and methylation levels in *Arabidopsis thaliana* plants obtained from seeds, somatic embryos and modified transformed plants: the modified plants derived from somatic embryos were found to demonstrate a higher level of variation in methylation (7.5%) compared to controls (3.2%) [31].

Our present metAFLP analysis using *Eco*RI/*Mse*I, *Kpn*I/*Mse*I and *Acc*65I/*Mse*I found a number of variations between the PSE, DSE and TSE regenerants and the donor plant. In total, 24 pairs of selective primers identified 1277 DNA fragments, of which 3.13% constituted polymorphic bands. This small number of obtained polymorphic bands could be explained by the way the cultures were conducted: the nonembryogenic and embryogenic callus cultures, somatic embryos and subsequent induction of secondary embryos were initiated by direct somatic embryogenesis from a single donor plant; this could, to a large extent, narrow the pool of variability among the in vitro regenerants. Gao et al. [40] reported similar findings in a study of *Fressia hybrida* cultures also initiated by somatic embryogenesis from a single donor plant to minimize existing heterozygosity or natural explant mutations; they conclude that the genetic and epigenetic variability observed in their regenerants was the result of tissue culture stress.

In the present study, the UPGMA and PCoA cluster analysis found that the donor plant and PSE, DSE and TSE regenerants formed distinct groups. Nevertheless, it should be noted that PSE regenerants obtained through the callus phase constituted a more diverse group, as did DSE regenerants obtained from PSE embryos by direct somatic embryogenesis. The most compact clusters were TSE regenerants, which were obtained from DSE embryos by direct somatic embryogenesis.

These findings correlate with the results of the AMOVA, which found that 21% of variance corresponded to the variability “between” groups of regenerants and 79% to variability “within” the groups. However, no such clear grouping of *Coffea arabica* regenerants obtained by direct (DSE) or indirect somatic embryogenesis (ISE) was revealed in a previous UPGMA analysis by Sanchez-Teyer et al. [50]. Based on an AFLP analysis of DNA stability in regenerants, their results indicate that the somatic embryogenesis induces rearrangements at the DNA level, and revealed discrepancies between the mechanisms involved in the process of indirect and direct somatic embryogenesis. In contrast, no such regenerant variation was observed in cultures of S*mallanthus sonchifolis* [51], *Olea europaea* [52] or *Coriandrum sativum* [53], despite the fact that the regeneration process also went through the callus phase.

In the present study, it is possible that, apart from growth regulators, the greater diversity in the PSE *P. filicifolia* embryos could be due to the relatively long cultivation time: a total of 88 passages were performed between callus initiation and the creation of the somatic embryos. Indeed, plants regenerated by callus phase in long-term cultures have shown high variability in previous studies; for example, numerous embryos with one cotyledon or multiple cotyledons were observed in *Quercus suber* culture [54].

Somaclonal variation has also been observed in somatic seedlings derived from long-term cell cultures of *Coffea arabica*, i.e., 11 and 27-month-old calli; although all plants derived from younger cell cultures, i.e., four months, exhibited normal phenotypes, the variation in the latter group affected almost all the regenerated plants. However, neither MSAP nor AFLP analyses of somatic seedlings indicated any changes on the genetic level. Similarly, no genetic or epigenetic change was demonstrated in *Miscanthus ×giganteus* during long-term shoot cultivation [55].

It should be noted that in long-term cultures, regenerants can demonstrate variation not only at the morphological or genetic level, but also at the epigenetic level. Studies of 12-month *Elaeis guineensi* suspension cultures obtained from callus tissue originated from zygotic embryos found that cell proliferation in vitro induced DNA hypermethylation in a time-dependent manner [56].

In addition, RAPD, REMAP (retrotransposon microsatellite amplified polymorphism) and MSAP analysis of *Humulus lupulus* plants grown in vitro for 2 years did not reveal differences between control (field cultivated) and treated plants (cultivated in vitro) at the genetic level, even after 12 cycles of micropropagation; however, variation was detected at the epigenetic level between plants collected from field cultivation and plants growing in vitro. Among all the plants from the in vitro culture group, almost 30% of the detected fragments demonstrated the same pattern of change [57]. The authors conclude that the variation detected at the epigenetic level is associated with in vitro culture conditions.

In turn, long-term secondary somatic embryogenesis cultures of *Theobroma cacao* were found to demonstrate lowered embryogenic potential [58]. Global DNA methylation levels appeared to increase in the older somatic embryos during long-term in vitro culture, and DNA methylation increased during SE expression. It was also found that aging embryos could regain lost embryogenic potential after treatment with 5-azaC at a certain concentration.

In the current study, analysis of the second group of DSE regenerants, i.e., those obtained by direct somatic embryogenesis from primary embryos (PSE), demonstrated relatively large diversity compared to the TSE group, i.e., those obtained by direct somatic embryogenesis from DSE.

Regeneration of *Psidium guajava* plants by direct somatic embryogenesis without callus phase was found to provide genetically stable material [59]. In turn, Rani and Raina [60] suggest that plants regenerated from meristems or by direct somatic embryogenesis maintain plant genetic integrity with the least risk of genetic variation. Moreover, Carra et al. [61] report obtaining homogeneous plant material from *Anthurium andraeanum* cv. Fantasia by direct somatic embryogenesis. Furthermore, Gao et al. [41] report a much higher level of genetic variation in *Fresia hybrida* plants obtained by direct (0.97%) rather than indirect somatic embryogenesis (0.27%), based on the AFLP method. In turn, MSAP analysis revealed changes in cytosine DNA methylation in both CG and CNG levels compared to the donor plant. The authors suggest that tissue culture induces stress that may cause some hereditary epigenetic variations in flowering plants. In addition, *Coffea arabica* plants regenerated by somatic embryogenesis were found to demonstrate very high similarity with the mother plants, suggesting that the process of somatic embryogenesis is genetically stable and has a mechanism for selecting competent cells [62].

In the present study, the second aim was to compare the biochemical variation of the triterpenoid compounds expressed by in vitro and ex vitro TSE with that of nonembryogenic, embryogenic callus tissue, as well as that of the donor plant. Previous phytochemical studies of *Polyscias* spp. have mainly focused on the isolation and identification of the structure of isolated triterpene saponins and the identification of their aglycones. Further, production of phenolic acids as well as oleanolic acid in shoot cultures of *P. filicifolia* was studied [3,10,63]; however, UA and HE accumulation was not investigated yet.

As oleanolic acid is not present in its free state, the most efficient extraction conditions were determined. Previous studies indicate that both the type of solvent used for extraction and the method of extraction play a key role in the final assessment of the quality and quantity of the tested plant material [64,65,66,67]. The results indicating that methanol was definitely the most suitable extractant are consistent with Janicsák et al. [68]. Methanol has frequently been used as an extractant of triterpene saponins and/or their aglycones [65,68,69,70,71,72]. In turn, Yin et al. [73] reported that ethyl acetate was the most efficient extractant for triterpene acids in three types of *Cyclocarya paliurus* plant tissue, while Marchev et al. [74] indicated that acetone provided the most efficient extraction of triterpene acids in *Salvia scabiosifolia* leaf and callus.

In the present study, extraction using a Soxhlet apparatus was found to yield higher recovery of investigated triterpenes than sonication. This superiority has been demonstrated in various tissues and plants by Janicsák et al. [64,68], and Wójciak-Kosior et al. [67]. However, a study of various approaches by Wójciak-Kosior et al. [67] found the most efficient method to be the use of a heat reflux condenser, while Soxhlet extraction provided slightly less performance.

Our present findings indicate that OA predominated among the investigated aglycones. Its yield was highest in TSE cultivated in vitro, and the lowest was in callus tissue; this difference may be due to the fact that not all the biosynthesis pathways of the compounds had been activated in the callus. It was previously reported that the higher productivity of plant in vitro cultures is connected with differentiation of cultivated cells reviewed in [75]. This phenomenon was also described in respect to OA glycosides accumulation (expressed as the quantity of aglycone glycosides after hydrolysis) in in vitro cultures of *Calendula officinalis.* In undifferentiated cell suspension cultures, the OA content amounted to 0.06 mg/g DW and increased up to 0.84 mg/g DW under treatment with 100 µM of methyl jasmonate [69]. While in hairy root cultures even the initial level of OA was higher and ranged from 4.59 to 8.42 mg/g DW depending on root line [70]. Further, OA content was significantly augmented in *C. officinalis* hairy root cultures from 2.56 or 3.75 mg/g DW according to the root line to 52.52 and 41.18 mg/g DW, respectively, when elicitation with 100 µM of jasmonic acid was applied [76]. In addition, higher content of investigated compounds in TSE cultivated in vitro then ex vitro could be explained by the fact that artificial conditions act as a stressor which could induce secondary metabolism. Nevertheless, in studies of *Cyclocarya paliurus*, higher total tritepenic acid (TTA, i.e., OA, UA and betulic acid) content was found, in suspension culture (6.24%) rather than in callus (2.16%) or in the leaves of soil-grown plants (3.74%) [73].

The results of the current study demonstrated that the somatic embryogenesis route could be efficiently applied to production of genetically stable *Polyscias filicifolia* plants capable of biosynthesizing triterpene compounds.

## 4. Materials and Methods

### 4.1. Plant Material and In Vitro Culture Conditions

The following plant material was subjected to morphological and molecular analysis: one donor plant of *Polyscias filicifolia* (R) and three groups of 15 regenerants collected from ^1^/_3_ strength MS [77] hormone-free solid medium. A voucher specimen of *P. filicifolia* was deposited at the Department of Pharmaceutical Biology and Medicinal Plants Biotechnology, Faculty of Pharmacy, Medical University of Warsaw, Poland (accession No. FW21/026/1999).

The first experimental group of plants for molecular research comprises plantlets of *P. filicifolia* (i) obtained through indirect primary somatic embryogenesis (PSE). Briefly, PSE were obtained by induction of nonembryogenic callus on 7-day-old leaves, isolated from 2-year-old donor plant on MS solid medium supplemented with 2 mg/L dichlorophenoxyacetic acid (2,4-D) and 2 mg/L 6-benzylaminopurine (BAP), followed by transferring onto medium containing 2 mg/L 2,4-D and 0.01 mg/L kinetin (KIN; N^6^-furfuryladenine) to induce embryogenic callus. The resulting friable callus was put into hormone-free ½ strength MS (½ MS) liquid medium where different stages of primary somatic embryos were developed though four 6-week passages. For the embryo germination, NN medium [78] supplemented with 0.1 mg/L indole-3-butyric acid (IBA) and 10 mg/L adenine sulfate (Ads) was used. After four weeks of culture, 15 randomly selected plantlets (PSE) were collected for molecular analysis.

The second experimental group (ii) secondary somatic embryos (DSE) were induced on the hypocotyls (about 2–4 mm) of seven-day-old primary somatic embryos (PSE) on hormone-free ½ MS solid medium containing 15 g/L sucrose. For the conversion into plantlets, DSE were isolated and transferred on NN solid medium supplemented with 0.5 mg/L KIN, 0.1 mg/L IBA and 10 mg/L Ads and cultivated for one month. After that, 15 randomly selected plantlets were collected for morphological and molecular analysis.

The third experimental group (iii) comprised plantlets obtained through direct somatic embryogenesis from DSE (TSE). The TSE were induced at the hypocotyl region of DSE cultivated on hormone-free ½ MS solid medium for four weeks. Subsequently, 15 randomly selected plantlets were isolated and collected for morphological and molecular analyses. The method of regenerating *Polyscias filicifolia* regenerants for genetic testing is presented in Figure S1. The detailed protocols for development of PSE and DSE regenerants were described previously by Śliwińska et al. [3]. The following morphological features of the regenerants were compared: shoot length, number of leaves, average length of the largest leaf and number of roots.

Microscopic assessment of PSE, DSE and TSE somatic embryos was carried out using an Olympus IIILC 2 stereoscopic optical microscope.

### 4.2. Amplified Fragment Length Polymorphism (AFLP) Analysis

#### 4.2.1. DNA Extraction

Total genomic DNA was extracted from approximately 100 mg of leaves from the donor plant and 45 regenerants: PSE, DSE and TSE following the procedure suggested by the manufacturer of the DNeasy Plant Mini Kit (Qiagen, Hilden, Germany). DNA integrity was analyzed electrophoretically in 1.4% agarose gel containing 1 × TBE buffer and ethidium bromide (0.5 µg/mL) under 20 V/cm. DNA concentration was estimated using a U*V/V*IS spectrophotometer (GeneQuanta, Pharmacia LKB).

#### 4.2.2. AFLP Analysis

The AFLP technique was performed according to the procedure described by Vos et al. [79] with some modifications according to Bednarek et al. [26]. A 500 ng of DNA was restricted with 10 U of *Eco*RI and 3 U of *Mse*I (New England Biolabs, St. Ipswich MA, USA) in 1 × ligation buffer (New England Biolabs) containing 50 mM NaCl and 5 µg BSA. This step was carried out in 10 µL at 37 °C for 3 h. After digestion, the enzymes were inactivated at 70 °C for 15 min following ligation of the heteroduplexes called adapters (Table S1) to sticky ends of the digested DNAs. The ligation step was carried out in 25 µL at 37 °C for 24 h. The ligation mixture consisted of 10 µL of the digested sample, to which 5 pmol of *Eco*RI and 50 pmol of *Mse*I adapters, 1 U of T4 DNA ligase, in 50 mM NaCl, 12.5 µg BSA, 1 × ligation buffer (New England Biolabs) were added. Obtained products were diluted in a 1 × TE buffer (1:6). A preselective amplification step followed this in a total volume of 25 µL. The reaction mixture consisted of 2.5 µL of the 1:6 diluted template, 1 × PCR buffer, 2.5 mM MgCl_2_, 0.4 mM dNTPs, 1U Taq DNA polymerase (Qiagen) and 12 pM of each preselective primer (Table S1) using the following temperature profile: [94 °C for 30 s; 56 °C for 60 s; at 72 °C for 60 s] × 20 [4 °C −ꝏ]. Amplification products were diluted in 1 × TE (1:20). The second selective amplification by PCR was performed with eight primer pairs of *Eco*RI + 3 bases (^32^P labeled) and *Mse*I + 3 bases (*E*-ACT/*M*-CGA, *E*-ATC/*M*-CGA, *E*-ACG/*M*-CTT, *E*-AGG/*M*-CTT, *E*-AAA/*M*-CTG, *E*-ACA/*M*-CAC, *E*-AAG/*M*-CAC, *E*-AAC/*M*-CAC). The reactions were running in 10 µL in the presence of 0.5 µL of 5’-end-labeled (^32^P) *E*-XYZ (labeling reactions were performed in 50 µL in T4 polynucleotide kinase buffer, containing 0.8 µCi (γ^32^P)ATP, 10 U T4 polynucleotide kinase at 37 °C for 60 min), 0.5 µL (50 pM) of unlabeled *M*-XYZ selective primers, 1.5 µL of the 1:20 diluted template, in 1 × PCR buffer, 2.5 mM MgCl_2_, 0.4 mM dNTP and 0.02 U of HotStarTaq DNA polymerase (Qiagen). The following cycling parameters were utilized for selective amplifications: [94 °C for 4 min][94 °C for 30 s; 65 °C^Ramp 0.7 °C^ for 30 s; 72 °C for 60 s] × 13 [94 °C for 30 s; 56 °C for 30 s; 72 °C for 60 s] × 30, [72 °C for 60 s] [4 °C −ꝏ]. PCR amplified DNA products were denatured at 90 °C for 5 min following immediate cooling on ice in the presence of 6 µL of the denaturing dye (98% formamide, 2% 0.5 M EDTA, 0.05% bromophenol blue and 0.05% xylene cyanol). Aliquots (6 µL) of sample solutions were loaded onto denaturing 7% polyacrylamide gel following electrophoretic separation on PAGE gel and exposition to X-ray film (FOTON XC) at −70 °C for 24–48 h.

#### 4.2.3. metAFLP Analysis

To detect epigenetic changes among donor plants (R) of *P. filicifolia* and three groups of PSE, DSE, TSE regenerants, metAFLP was carried out according to the method described by Bednarek et al. [26]. The list of metAFLP adapters, primers (preselective and selective primer pair combinations) sequences is given (Table S1). Briefly, 500 ng of genomic DNA representing control (donor plant) and PSE, DSE and TSE regenerants was digested with *Acc*65I/*Mse*I and another aliquot of the same genomic DNA was treated with *Kpn*I/*Mse*I endonucleases. Eight selective primer combinations were applied: CpXpG-AAG/*M*-CGC; CpG-TGA/*M*-CGC; CpG-GAG/*M*-CGA; CpG-GGT/*M*-CGT; CpG-GGT/*M*-CGC; CpXpG-AGT/*M*-CTG; CpG-GAG/*M*-CAC; CpXpG-AGG/*M*-CAC for each pair of enzymes. Following adapter ligation, preselective and selective PCR with ^32^P labeled primer amplifications were performed as described above. PCR products were denatured and separated on a 7% denaturing polyacrylamide gel following an overnight X-ray film exposure at −70 °C.

#### 4.2.4. Marker Analysis

The GeneAlEx 6.5b5 program [80] was used for the characterization of markers (marker frequencies, Shannon’s *I*—index, polymorphisms, etc.). Analysis of Molecular Variance (AMOVA) was applied to differentiate tested groups of regenerants using GeneAlEx software. The estimates of similarity were based on Dice coefficient and clustering was performed using the Ward clustering method. Principal Component Analysis (PCA), Principal Coordinate Analysis (PCoA) and Analysis of Variance (ANOVA) were conducted in Statistica PL 13.3.

### 4.3. Phytochemical Analysis

All reagents were purchased from Avantor Performance Materials (Gliwice, Poland) and Sigma-Aldrich (Poznań, Poland).

#### 4.3.1. Preparation of Plant Extracts

The following plant material was extracted: nonembryogenic and embryogenic callus, TSE plantlets cultivated in vitro and ex vitro (Figure S2). First, plant material 1.0 ± 0.002 g was extracted with petroleum ether (50 mL 4× for 30 min) in an ultrasonic bath (Sonorex Bandelin RK 100, Berlin, Germany). The resulting extracts were evaporated and extracted with methanol, acetone or ethyl acetate using an ultrasonic bath. In addition to the above described procedures, TSE plantlets were also subjected to extraction in Soxhlet apparatus for 6 h according to the method described by Janicsák et al. [64].

#### 4.3.2. Isolation of the Saponin Fraction

The evaporated dry methanol, acetone or ethyl acetate extracts were dissolved in 100 mL distilled water and extracted 4× with 50 mL n-butanol saturated with distilled water. After evaporation, the dry residue constituted a raw saponin fraction. The evaporated to dryness butanol extracts were then dissolved in 5 mL of methanol and subjected to hydrolysis with 50 mL of Kiliani mixture in a boiling water bath at reflux for 5 h at 80 °C. The hydrolysates were diluted 1:3 (*v/v*) with distilled water, filtered and extracted four times with diethyl ether using 50 mL of solvent each time. The combined ether extracts were rinsed with cold distilled water to bring the organic layer to pH = 5.5 and then evaporated to dryness.

##### Purification of the Ether Extract by SPE Method

Sample purification was performed by solid phase extraction (SPE) according to the method presented by [35]. Chromabond SB (Macherey-Nagel, Düren, Germany) polypropylene columns with a bed weight of 500 mg and a capacity of 6 mL were used. The dry extracts were dissolved in 2 mL methanol. Then, 0.5 mL was taken and evaporated to dryness under a stream of nitrogen and next redissolved in 2 mL of methanol, further mixed with 8 mL of deionized water to give a solution of the sample in 20% methanol. The samples were applied to SPE columns and washed with 5 mL of deionized water under normal pressure. Residual water was removed under reduced pressure. Aglycones were eluted from the columns into reaction vessels with 5 mL methanol and evaporated to dryness under a stream of nitrogen.

##### Silylation of Samples

The saponin aglycones present in the SPE eluate were silylated according to the method described by Razboršek et al. [81]. First, 100 μL of a solution of cholesterol acetate (CA; Sigma-Aldrich, Poland) in methanol (0.13 mg/mL) was added to the reaction vessels. The solvent was evaporated to dryness under a nitrogen stream. CA was added to control the volume of injected sample (injection standard). Then, 50 µL of anhydrous pyridine and 100 µL of N-methyl-N-tri-methylsilyltrifluoroacetamide were added and the vessels were put into a dry 1 bath (Grant QBT) at 80 °C for 2 h.

#### 4.3.3. Determination of Oleanolic and Ursolic Acid and Hederagenin in Analytical Samples by GC-FID

For identification and quantification of oleanolic acid (OA), ursolic acid (UA) and hederagenin (HE) in analytical samples, commercial analytical standards of these compounds (≥97%) were purchased from Sigma-Aldrich (Poland) to perform method validation. In volumetric flasks (10 mL), methanol stock solutions of reference substances OA, UA and HE and internal standard CA were prepared with concentrations of 0.1 mg/mL for OA, 0.12 mg/mL for UA, 0.11 mg/mL for HE and 0.13 mg/mL for CA. After evaporation of the samples to dryness in a stream of nitrogen, silylation was carried out as described above. First, 1 µL of silylated standard solutions was applied to the GC column. Chromatographic analysis was performed using GC-17A gas chromatograph (Shimadzu, Kyoto, Japan) instrument equipped with flame ionization detector (FID) and fitted with a 30 m × 0.32 mm × 0.25 µm Optima 5 capillary column (Macherey-Nagel, Germany) ID. Analysis parameters were as follows: carrier gas: helium (He) with a flow rate of 0.4 mL/s; injector temperature: 290 °C, detector temperature 300 °C. The initial oven temperature was 200 °C for 1 min; thereafter increased by 7 °C/min to 290 °C and held for 1 min and increased by 10 °C/min to 320 °C and held for 35 min; total time of analysis was 52.9 min. Retention times of internal standards of examined aglycones were established in the presence of the internal standard—CA of retention time 20.255 min. Retention times of trimethylsilylated reference substances were 25.280 min for OA; 26.163 min for UA; 27.345 min for HE, respectively. External calibration curves were prepared by triplicate injection of each of seven dilutions of OA, UA and HE methanol solutions of different concentration to the chromatographic column and performing GC-FID analysis, as described above. To check the linearity, the curves of the peak area dependence on the concentration were plotted and the curve equations and determination coefficients (R^2^) were calculated. For recovery test for OA and HE, reference substances at concentration of 500 and 800 µL OA and 350 µL HE were applied to SPE columns. The substances were eluted and then silylated as described above. The determinations were repeated six times. The results are presented as standard deviation and coefficient of variation (CV%) (Table S2).

For identification of trimethylsilylated derivatives of OA, UA and HE in analytical samples, retention times of peaks present in analytical samples were compared with the retention times of analytical standards, and for quantification of OA, UA and HE, content calibration curves were used. Results were expressed as μg of each aglycone per μL of analytical sample and calculated into μg of each aglycone per one g of dry weight (DW) of plant material [μg/g DW].

All experiments were carried out in triplicate and the statistical significance between means was assessed using the ANOVA analysis of variance performed with STATISTICA 13.1 PL software. A probability of *p* < 0.05 was considered significant.

## Figures and Tables

**Table 1:** Summary of morphological features of three groups of *Polyscias filicifolia* regenerants obtained by indirect and direct somatic embryogenesis.

Set of Regenerants	Shoot Length [cm]	Number of Leaves	The Average Length of the Largest Leaf [cm]	Number of Roots
PSE	7.4 ± 0.36 *	7.5 ± 0.70	1.78 ± 0.40	13.6 ± 1.80
DSE	4.7 ± 0.22 *	7.6 ± 0.52	1.68 ± 0.27	13.2 ± 1.62
TSE	3.55 ± 0.2 *	6.2 ± 0.36 *	0.76 ± 0.06 *	10.8 ± 0.83 *

PSE—plantlets regenerated through indirect somatic embryogenesis; DSE—plantlets regenerated through direct somatic embryogenesis from PSE; TSE—plantlets regenerated through direct somatic embryogenesis from DSE; presented values are means (N = 15) ± SD, values marked with asterisk within groups are statistically different (*p* < 0.05) from the others.

**Table 2:** Distribution of AFLP and metAFLP fragments in three groups of regenerants and donor *Polyscias filicifolia* plant.

**AFLP Fragments**									
**No**	**Selective Primers**	**Total NUMBER of Bands**		**Polymorphic Bands**		**Monomorphic Bands**		**Percentage of Polymorphic Bands**	
1	*E*-ACT/*M*-CGA	61		1		60		1.64	
2	*E*-ATC/*M*-CGA	68		3		65		4.41	
3	*E*-ACG/*M*-CTT	48		0		48		0	
4	*E*-AGG/*M*-CTT	62		1		61		1.61	
5	*E*-AAA/*M*-CTG	41		0		41		0	
6	*E*-ACA/*M*-CAC	51		0		51		0	
7	*E*-AAG/*M*-CAC	34		0		34		0	
8	*E*-AAC/*M*-CAC	63		0		63		0	
	∑	428		5		423		1.17	
**metAFLP Fragments**									
**No**	**Selective Primers**	**Total Number of Bands**		**Polymorphic Bands**		**Monomorphic Bands**		**Percentage of Polymorphic Bands**	
		***Kpn*** **I/*Mse*I**	***Acc*** **65I/*Mse*I**	***Kpn*** **I/*Mse*I**	***Acc*** **65I/*Mse*I**	***Kpn*** **I/*Mse*I**	***Acc*** **65I/*Mse*I**	***Kpn*** **I/*Mse*I**	***Acc*** **65I/*Mse*I**
1	CpXpG-AAG/M-CGC	66	65	8	1	58	64	12.12	1.54
2	CpG-TGA/M-CGC	52	50	0	3	52	47	0	6.00
3	CpG-GAG/M-CGA	52	52	4	2	48	50	7.69	3.85
4	CpG-GGT/M-CGT	54	54	3	3	51	51	5.56	5.56
5	CpG-GGT/M-CGC	53	54	3	3	50	51	5.66	5.56
6	CpXpG-AGT/M-CTG	39	43	2	3	37	40	5.13	6.98
7	CpG-GAG/M-CAC	52	52	0	0	52	52	0	0
8	CpXpG-AAG/M-CAC	54	57	0	0	54	57	0	0
∑		422	427	20	15	402	412	4.74	3.51

**Table 3:** Number of specific bands identified between three groups of regenerants using the AFLP and metAFLP methods.

Method	AFLP			metAFLP					
Restriction Enzyme Pair	*Eco*RI/*Mse*I			*Kpn*I/*Mse*I			*Acc*65I/*Mse*I		
Group of regenerants	PSE	DSE	TSE	PSE	DSE	TSE	PSE	DSE	TSE
Total number of bands	427	427	427	422	422	418	425	426	425
Number of specific bands	0	0	0	2	0	0	0	1	1

**Table 4:** Number of specific bands identified in the electrophoretic profiles between three sets of *Polyscias filicifolia* regenerants and the donor plant using three restriction enzyme pairs.

Method	AFLP		metAFLP			
Restriction Enzyme Pair	*Eco*RI/*Mse*I		*Kpn*I/*Mse*I		*Acc*65I/*Mse*I	
Group of regenerants	PSE/DSE/TSE	Donor plant	PSE/DSE/TSE	Donor plant	PSE/DSE/TSE	Donor plant
Total number of bands	428	425	422	416	427	419
Number of specific bands	3	0	6	0	8	0

**Table 5:** The effect of solvent type and method of extraction on the yield of triterpene aglycones [µg/g DW] in *Polyscias filicifolia* TSE regenerants cultured in vitro.

Plant Material	Oleanolic Acid	Ursolic Acid	Hederagenin	Ratio OA to HE
Nonembryogenic callus	18.62 ± 0.28 ^a^	not detected	9.53 ± 0.50 ^a^	(2:1)
Embryogenic callus	17.64 ± 0.15 ^b^	not detected	9.81 ± 0.33 ^b^	(2:1)
TSE regenerants	439.72 ± 0.64 ^a,b,c^	19.07 ± 0.21 ^a^	111.85 ± 0.48 ^a,b,c^	(4:1)
TSE plants ex vitro	47.10 ± 0.87 ^a,b,c^	8.82 ± 0.55 ^a^	16.06 ± 0.39 ^a,b,c^	(3:1)
Donor plant	55.37 ± 0.94 ^a,b,c^	7.90 ± 0.41 ^a^	17.02 ± 0.58 ^a,b,c^	(3:1)

Data of oleanolic acid (OA), ursolic acid (UA) and hederagenin (HE) were expressed by mean ± SD values. Mean values denoted with same the same letter within groups were significantly different at *p* ˂ 0.05 according to Tukey test. OA: oleanolic acid; UA: ursolic acid; HE: Hederagenin; SD: Standard deviation; DW: Dry weight.

**Table 6:** The content of three triterpene aglycones [µg/g DW] in different plant material of *Polyscias filicifolia* cultivated in vitro and ex vitro.

Extraction Solvent				
	Oleanolic Acid	Ursolic Acid	Hederagenin	Ratio OA to HE
Acetone	186.76 ± 0.60 ^a^	9.46 ± 0.13 ^a^	17.98 ± 0.56 ^a^	(10:1)
Methanol	307.22 ± 0.49 ^a,b^	18.07 ± 0.24 ^a,b^	34.01 ± 0.42 ^a,b^	(9:1)
Ethyl acetate	98.72 ± 0.24 ^a,b^	7.73 ± 0.49 ^b^	14.51 ± 0.44 ^b^	(7:1)
**Extraction Method with Methanol**				
Sonification	307.22 ± 0.49 ^a^	18.07 ± 0.24 ^a^	57.07 ± 0.64 ^a^	(5:1)
Soxhlet	439.72 ± 0.23 ^a^	28.09 ± 0.56 ^a^	111.34 ± 0.67 ^a^	(4:1)

Data of OA, UA and HE were expressed by mean ± SD values. Mean values denoted with the same letter within groups were significantly different at *p* ˂ 0.05 according to Tukey test. OA: oleanolic acid; UA: ursolic acid; HE: hederagenin; SD: standard deviation; DW: dry weight.

## Data Availability

Data are contained within the article and supplementary material.

## Supplementary Materials

The following are available online at https://www.mdpi.com/article/10.3390/ijms22115752/s1.
