# Supplementary material for: Comparative Study of the Genetic and Biochemical Variability of Polyscias filicifolia (Araliaceae) Regenerants Obtained by Indirect and Direct Somatic Embryogenesis as a Source of Triterpenes
Source: Int J Mol Sci. 2021 May 27;22(11):5752. doi: 10.3390/ijms22115752 (PMC8198449; doi:10.3390/ijms22115752)
Supplement: Supplementary file 1 [file ijms-22-05752-s001.zip › Figure S2.pdf]

Comparative study of the genetic and biochemical variability of *Polyscias filicifolia* (Araliaceae) regenerants obtained by indirect and direct somatic embryogenesis as a source of triterpenes

Anita A. Śliwińska [1], Agnieszka Białek [2], Renata Orłowska [3], Dariusz Mańkowski [3], Katarzyna Sykłowska-Baranek [1]* and Agnieszka Pietrosiuk [1]

[1] Department of Pharmaceutical Biology and Medicinal Plant Biotechnology, Faculty of Pharmacy, Medical University of Warsaw, Banacha 1, 02-097 Warsaw, Poland; asliwinska@wum.edu.pl (AŚ); katarzyna.syklowska-baranek@wum.edu.pl (KSB); agnieszka.pietrosiuk@wum.edu.pl (AP)
[2] Department of Biotechnology and Nutrigenomics, Institute of Genetics and Animal Biotechnology, Polish Academy of Sciences, Postępu 36 A, Jastrzębiec, 05-552 Magdalenka, Poland; a.bialek@ighz.pl (AB)
[3] Plant Breeding and Acclimatization Institute-National Research Institute, Radzików, 05-870 Błonie, Poland; r.orlowska@ihar.edu.pl (RO); d.mankowski@ihar.edu.pl (DM)
* Correspondence: katarzyna.syklowska-baranek@wum.edu.pl (KSB)

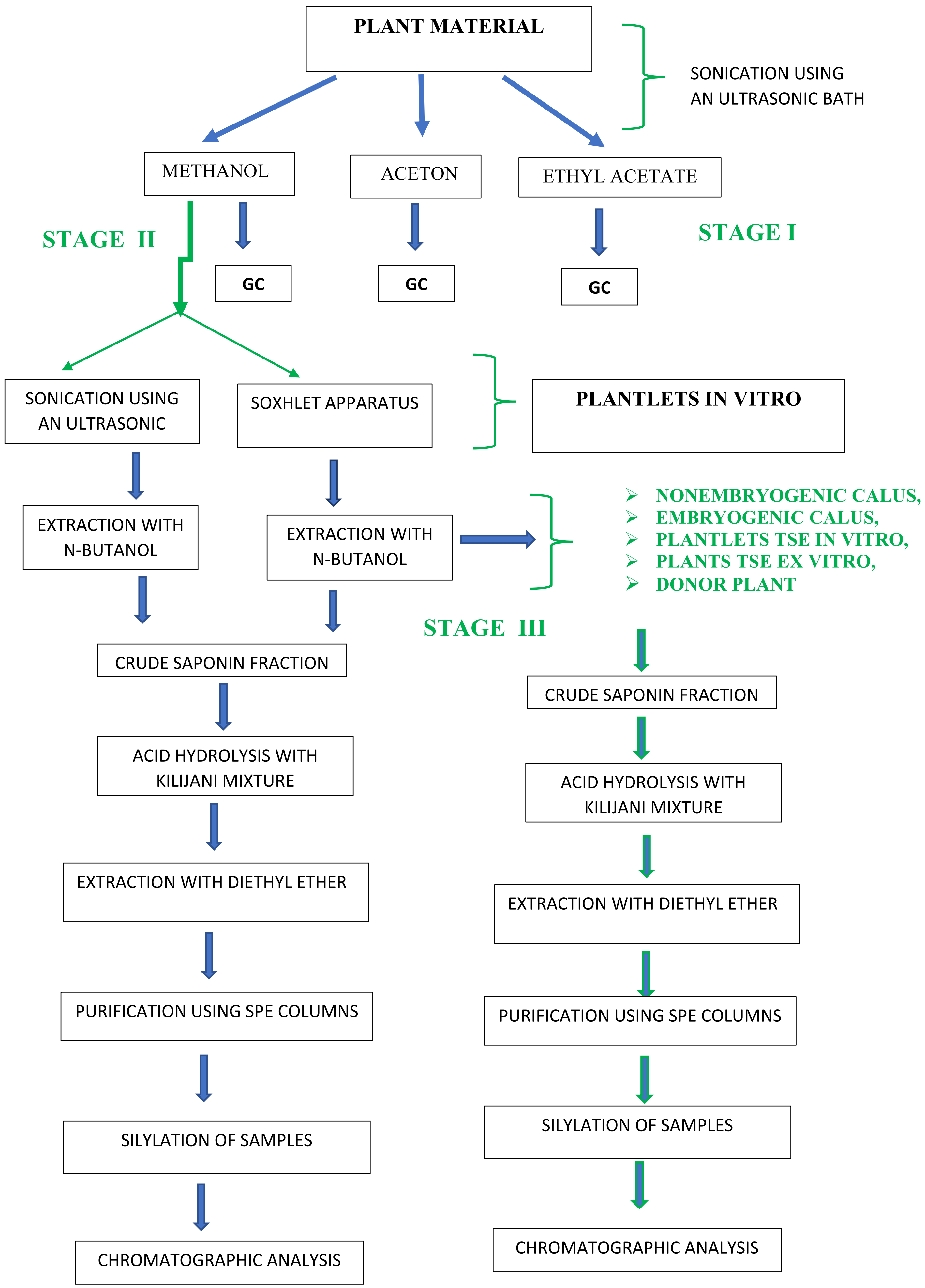
PLANT MATERIAL
SONICATION USING AN ULTRASONIC BATH
METHANOL
ACETON
ETHYL ACETATE
STAGE II
STAGE I
GC
GC
GC
SONICATION USING AN ULTRASONIC
SOXHLET APPARATUS
PLANTLETS IN VITRO
EXTRACTION WITH N-BUTANOL
EXTRACTION WITH N-BUTANOL
NONEMBRYOGENIC CALUS,
EMBRYOGENIC CALUS,
PLANTLETS TSE IN VITRO,
PLANTS TSE EX VITRO,
DONOR PLANT
STAGE III
CRUDE SAPONIN FRACTION
CRUDE SAPONIN FRACTION
ACID HYDROLYSIS WITH KILIJANI MIXTURE
ACID HYDROLYSIS WITH KILIJANI MIXTURE
EXTRACTION WITH DIETHYL ETHER
EXTRACTION WITH DIETHYL ETHER
PURIFICATION USING SPE COLUMNS
PURIFICATION USING SPE COLUMNS
SILYLATION OF SAMPLES
SILYLATION OF SAMPLES
CHROMATOGRAPHIC ANALYSIS
CHROMATOGRAPHIC ANALYSIS

Figure S2. The scheme of procedure of plant material extraction and sample preparation
