# Supplementary material for: Comparative Study of the Genetic and Biochemical Variability of Polyscias filicifolia (Araliaceae) Regenerants Obtained by Indirect and Direct Somatic Embryogenesis as a Source of Triterpenes
Source: Int J Mol Sci. 2021 May 27;22(11):5752. doi: 10.3390/ijms22115752 (PMC8198449; doi:10.3390/ijms22115752)
Supplement: Supplementary file 1 [file ijms-22-05752-s001.zip › Table S1.pdf]

Comparative study of the genetic and biochemical variability of *Polyscias filicifolia* (Araliaceae) regenerants obtained by indirect and direct somatic embryogenesis as a source of triterpenes

Anita A. Śliwińska [1], Agnieszka Białek [2], Renata Orłowska [3], Dariusz Mańkowski [3], Katarzyna Sykłowska-Baranek [1*] and Agnieszka Pietrosiuk [1]

[1] Department of Pharmaceutical Biology and Medicinal Plant Biotechnology, Faculty of Pharmacy, Medical University of Warsaw, Banacha 1, 02-097 Warsaw, Poland; asliwinska@wum.edu.pl (AŚ); katarzyna.syklowska-baranek@wum.edu.pl (KSB); agnieszka.pietrosiuk@wum.edu.pl (AP)

[2] Department of Biotechnology and Nutrigenomics, Institute of Genetics and Animal Biotechnology, Polish Academy of Sciences, Postępu 36 A, Jastrzębiec, 05-552 Magdalenka, Poland; a.bialek@ighz.pl (AB)

[3] Plant Breeding and Acclimatization Institute-National Research Institute, Radzików, 05-870 Błonie, Poland; r.orlowska@ihar.edu.pl (RO); d.mankowski@ihar.edu.pl (DM)

* Correspondence: katarzyna.syklowska-baranek@wum.edu.pl (KSB)

Table S1. Adapter and primer sequences

Adapter/Primer	Sequence (5' - 3')
*Eco*RI adapter 1	CTC GTA GAC TGC GTA CC
*Eco*RI adapter 2	CAT CTG ACG CAT GGT TAA
*Mse*I adapter 1	TAC TCA GGA CTC ATA
*Mse*I adapter 2	GAG TCC TGA GTA GCA G
*Kpn*I adapter 1	CTC GTA GCA TGC GTA CAG TAC
*Kpn*I adapter 2	TGT ACG CAT GCT AC
*Acc*65I adapter 1	CTC GTA GCA TGC GTA CA
*Acc*65I adapter 1	GTA CTG TAC GCA TGC TAC
*Eco*RI pre-selective primer	GAC TGC GTA CCA ATT CA
*Mse*I pre-selective primer	GAT GAG TCC TGA GTA AAC
*Acc*65I/*Kpn*I pre-selective primer	GCA TGC GTA CAG TAC C
*Eco*RI selective primer	GAC TGC GTA CCA ATT CAxy
*Mse*I selective primer	GAT GAG TCC TGA GTA AACxy
*Acc*65I/*Kpn*I selective primer	GCA TGC GTA CAG TAC Cxy

xy- means two arbitrary nucleotides
