# Supplementary material for: Comparative Study of the Genetic and Biochemical Variability of Polyscias filicifolia (Araliaceae) Regenerants Obtained by Indirect and Direct Somatic Embryogenesis as a Source of Triterpenes
Source: Int J Mol Sci. 2021 May 27;22(11):5752. doi: 10.3390/ijms22115752 (PMC8198449; doi:10.3390/ijms22115752)
Supplement: Supplementary file 1 [file ijms-22-05752-s001.zip › Table S3.pdf]

Comparative study of the genetic and biochemical variability of *Polyscias filicifolia* (Araliaceae) regenerants obtained by indirect and direct somatic embryogenesis as a source of triterpenes

Anita A. Śliwińska [1], Agnieszka Białek [2], Renata Orłowska [3], Dariusz Mańkowski [3], Katarzyna Sykłowska-Baranek [1]* and Agnieszka Pietrosiuk [1]

[1] Department of Pharmaceutical Biology and Medicinal Plant Biotechnology, Faculty of Pharmacy, Medical University of Warsaw, Banacha 1, 02-097 Warsaw, Poland; asliwinska@wum.edu.pl (AŚ); katarzyna.syklowska-baranek@wum.edu.pl (KSB); agnieszka.pietrosiuk@wum.edu.pl (AP)
[2] Department of Biotechnology and Nutrigenomics, Institute of Genetics and Animal Biotechnology, Polish Academy of Sciences, Postępu 36 A, Jastrzębiec, 05-552 Magdalenka, Poland; a.bialek@ighz.pl (AB)
[3] Plant Breeding and Acclimatization Institute-National Research Institute, Radzików, 05-870 Błonie, Poland; r.orlowska@ihar.edu.pl (RO); d.mankowski@ihar.edu.pl (DM)
* Correspondence: katarzyna.syklowska-baranek@wum.edu.pl (KSB)

Table S3. Summary of metAFLP data for three groups of regenerants PSE, DSE, TSE of *Polysias filicifolia*

**Type of variation**	**% of variation**		
	**PSE**	**DSE**	**TSE**
Sequence variation	1.25	1.11	0.75
Demethylation	0.54	0.56	0.84
De novo methylation	0.41	0.25	0.18
Total	2.20	1.92	1.77
